# Expression of Human Endogenous Retrovirus-W Including Syncytin-1 in Cutaneous T-Cell Lymphoma

**DOI:** 10.1371/journal.pone.0076281

**Published:** 2013-10-01

**Authors:** Pilvi Maliniemi, Michelle Vincendeau, Jens Mayer, Oliver Frank, Sonja Hahtola, Leena Karenko, Emilia Carlsson, Francois Mallet, Wolfgang Seifarth, Christine Leib-Mösch, Annamari Ranki

**Affiliations:** 1 Department of Dermatology and Allergology, Helsinki University Central Hospital, University of Helsinki, Helsinki, Finland; 2 Institute of Virology, Helmholtz Zentrum München, German Research Center for Environmental Health, Neuherberg, Germany; 3 Department of Human Genetics, Center of Human and Molecular Biology, Medical Faculty, University of Saarland, Homburg, Germany; 4 Department of Hematology and Oncology, Mannheim Medical Center, University of Heidelberg, Mannheim, Germany; 5 Joint Unit Hospices Civils de Lyon-bioMérieux, Cancer Biomarkers Research Group, Centre Hospitalier Lyon Sud, Pierre Bénite, France; 6 Research Unit Cellular Signal Integration, Institute of Molecular Toxicology and Pharmacology, Helmholtz Zentrum München, German Research Center for Environmental Health, Neuherberg, Germany; Helmholtz Zentrum Muenchen - German Research Center for Environmental Health, Germany

## Abstract

The pathomechanism of mycosis fungoides (MF), the most common type of primary cutaneous T-cell lymphomas (CTCLs) and a malignancy of non-recirculating, skin-resident T-cells, is unknown albeit underlying viral infections have been sought for. Human endogenous retroviruses (HERVs) are ancient retroviral sequences in the human genome and their transcription is often deregulated in cancers. We explored the transcriptional activity of HERV sequences in a total of 34 samples comprising MF and psoriasis skin lesions, as well as corresponding non-malignant skin using a retrovirus-specific microarray and quantitative RT-PCR. To identify active HERV-W loci, we cloned the HERV-W specific RT-PCR products, sequenced the cDNA clones and assigned the sequences to HERV-W loci. Finally, we used immunohistochemistry on MF patient and non-malignant inflammatory skin samples to confirm specific HERV-encoded protein expression. Firstly, a distinct, skin-specific transcription profile consisting of five constitutively active HERV groups was established. Although individual variability was common, HERV-W showed significantly increased transcription in MF lesions compared to clinically intact skin from the same patient. Predominantly transcribed HERV-W loci were found to be located in chromosomes 6q21 and 7q21.2, chromosomal regions typically altered in CTCL. Surprisingly, we also found the expression of 7q21.2/*ERVWE1*-encoded Syncytin-1 (Env) protein in MF biopsies and expression of Syncytin-1 was seen in malignant lymphocytes, especially in the epidermotropic ones, in 15 of 30 cases studied. Most importantly, no Syncytin-1 expression was detected in inflammatory dermatosis (Lichen ruber planus) with skin-homing, non-malignant T lymphocytes. The expression of *ERVWE1* mRNA was further confirmed in 3/7 MF lesions analyzed. Our observations strengthen the association between activated HERVs and cancer. The study offers a new perspective into the pathogenesis of CTCL since we demonstrate that differences in HERV-W transcription levels between lesional MF and non-malignant skin are significant, and that *ERVWE1-*encoded Syncytin-1 is expressed in MF lymphoma cells.

## Introduction

CTCLs represent a group of heterogenous non-Hodgkin lymphomas arising mostly from CD4^+^ T-cells. The clinical behavior of these lymphomas varies from a non-progressive early mycosis fungoides (MF) to rapidly progressing leukaemic Sézary syndrome (SS) [1,2]. The most common type of CTCL, MF represents a malignancy of skin-homing T-cells, i.e. sessile, non-recirculating, skin-resident effector memory T-cells (T_EM_) [3]. Thus, the T-cell clone in the skin is infrequently found in peripheral blood and may differ from the one in the skin [4]. While CTCLs show a growing incidence, the underlying mechanisms are still largely unknown and no curative therapy exists so far.

Chromosomal instability is a typical feature of CTCL. Chromosome aberrations are diverse including numerical (DNA copy number changes) as well as structural (deletions, translocations, inversions) alterations. Frequently altered chromosomes include chromosomes 1, 2, 3, 6, 7, 8, 9, 10, 11, 12, 13, 14, 16, 17, and 19, but almost any chromosome can be involved [5-12].

With regard to CTCL pathogenesis, there are currently two main hypotheses. The antigen stimulation hypothesis suggests that persistent antigen stimulation leads to a continuous proliferation of T-cells and chronic inflammation, ultimately leading to the development of a malignant T-cell clone [13]. The second hypothesis suggests that a specific viral agent may serve as a triggering factor (antigen) [14]. Since MF, the most common form of CTCL, clinically resembles the smoldering form of adult T-cell lymphoma/leukemia, caused by human T-cell leukaemia/lymphoma virus I (HTLV-I) and since CTCL patients also have antibodies cross-reacting with HTLV-I and/or human immunodeficiency virus type 1 (HIV-1) core proteins [15,16] exogenous retroviruses were extensively sought for as an etiologic agent. However, neither replicating retroviruses nor any other viruses have been found in CTCL [17-22].

Human endogenous retroviruses (HERVs), on the other hand, represent a group of repetitive elements in the human genome. HERVs and related LTR-retroelements comprise about 8% of the human genome and at least HERVs stem from ancient retroviral infections of the germ cell genome. In primates, a major invasion and expansion of *pol* containing ERVs occurred after the New World Monkeys lineage separated from the Old World Monkeys and apes [23]. HERV elements, if they are full-length proviruses, comprise promoters and other transcription-regulatory elements within long-terminal-repeats (LTR), harbor genes for retroviral proteins (Gag, Pro, Pol, and Env) and some HERV groups even encode accessory proteins (for review, see 24 and HERVs and cancer [25-27].

Since initial germ line infection, HERVs amplified in copy numbers and almost all loci accumulated numerous mutations, thereby becoming coding-deficient [28,29]. Nevertheless, transcription of HERV elements may be re-activated e.g. by various environmental conditions such as chemicals, radiation or exogenous viruses [30-33]. In animals, recombination between different ERVs, or exogenous viruses and ERVs, resulted in novel pathogenic viruses causing leukemia and other tumors [34]. However, no infectious HERV has been detected in human. Instead, polymorphic HERVs, specifically presence/absence alleles of HERV proviral loci, have been reported (for a review, see 35.

Transcription of HERVs has been reported for all human tissues investigated so far, including healthy individuals [36-39]. Generally, HERVs are transcribed in a tissue-specific manner [40,41] and different cell types, each with a specific HERV transcription pattern, in a tissue are likely to determine the HERV transcription pattern of that tissue as a whole. Additionally, transcription of HERVs appears deregulated in cancers (for a review, see 25,42) and also inflammation may play a role in HERV activation. It is under debate though, whether inflammation is the cause or consequence of HERV activation [43-45]. For example, cytokines such as TNF-α are known to regulate HERV expression [46,47]. Although not proven, this may have affected the expression of a new endogenous retroviral variant found in psoriasis [48]. Moreover, functional regulatory sequences within HERV LTRs could affect transcriptional regulation of neighboring genes, e.g. activation of oncogenes or inactivation of tumor suppressor genes [25,49-53]. Notably, some HERVs still code for functional proteins, some of which might be associated with human diseases, like HERV-K (HML-2) encoded Rec or Np9 [25,52].

A locus of the human endogenous retrovirus group HERV-W has evolved to an essential gene (*ERVWE1*) that encodes Syncytin-1 and exerts important functions during placenta formation [54-56]. Syncytin-1 has also been implicated in the development of Multiple Sclerosis (MS) [57]. Besides Syncytin-1, sequences of unclear origin very similar to genomic HERV-W sequences and named Multiple sclerosis associated retrovirus (MSRV) have been reported [58]. Yet, the nature of those sequences was interpreted controversially [59].

We studied here the expression of HERVs in CTCL by investigating HERV transcription profiles and identifying active HERV loci in MF, in psoriasis skin lesions, and in corresponding, clinically intact skin of each patient. We further identified the transcribed HERV-W loci and examined HERV-W-derived Syncytin-1 expression both in MF and in non-malignant, inflammatory tissue samples.

## Results

The purpose of this investigation was to establish, for the first time, an overall endogenous retroviral transcription profile for MF skin and to evaluate the possible implications of differentially active retroviral elements in CTCL pathogenesis. Skin biopsies of psoriasis patients with no known malignant diseases served as a reference due to previously reported expression of HERV families HERV-W, K and E [48]. Of note, since HERVs show tissue-specific expression profiles (Table S1), and since MF, unlike Sezary syndrome, is a malignancy of sessile, non-recirculating skin-resident T-cells [4], only skin biopsies with such skin-resident lymphocytes were studied.

### A distinct, skin-specific HERV transcription profile identified in the skin of MF and psoriasis patients

**Figure 1:**
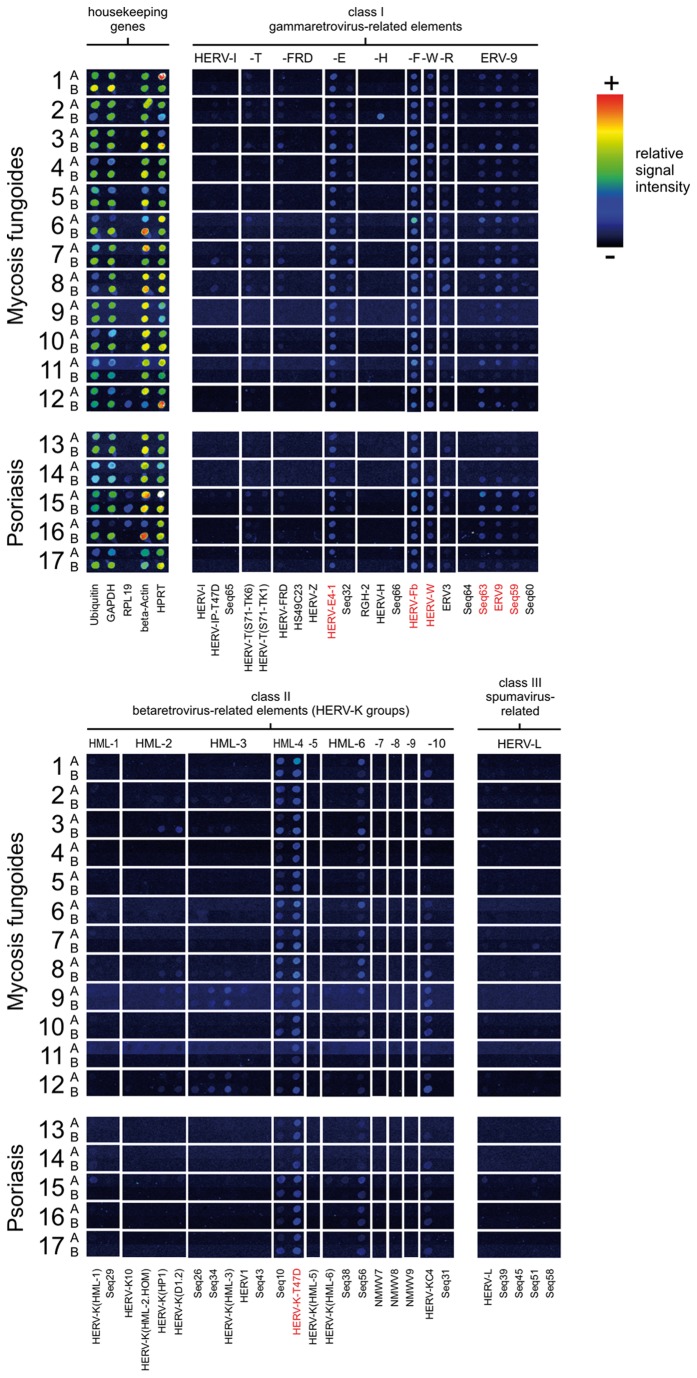
Retroarray analysis of HERV transcriptional activity in MF. HERV activity profiles representing pairs of lesion (A) and non-malignant (B) skin tissue specimens (digitally aligned). Each sample pair (n=17) was derived from an individual patient (MF 1-12, psoriasis 13-17). HERV subgroups representing a skin-specific core transcription profile (HERV-E, HERV-F, HERV-W, ERV-9, HERV-K(HML-4)) are emphasized with red letters. A panel of housekeeping genes (Ubiquitin, GAPDH, RPL19, β-Actin, HPRT) served as internal controls (for detailed information on methodology, see [77,79]). Each positive spot on the microarray may represent multiple HERV loci of one subgroup of multicopy HERV elements with sufficient sequence similarity that individual elements cannot be distinguished. Weak signals may be unrecognizable in the printed figure.

A retrovirus-specific microarray (RetroArray), previously described in detail [60], was used to define a distinct skin-specific HERV transcription profile [60]; see also Methods). In this semi-quantitative analysis, seven constitutively transcribed HERV taxa derived from five HERV groups (HERV-E, HERV-F, HERV-W, ERV9, HERV-K (HML-4) were detected in at least 90% of healthy skin samples (Figure 1). This HERV core transcription pattern was compared with the HERV core signature of other human tissues such as brain, mamma, urothelium, and kidney (see Table S1 for detailed information). In contrast to all other tissues investigated so far, primarily class I HERVs were found to be active in skin, whereas class II HERVs are less frequently transcribed. Since the array also contained probes specific for HTLV and HIV sequences transcripts from such retrovirus sequences should have been detected if present. Yet, all cases tested negative (data not shown).

In order to search for disease-related HERV activity, we then compared the signatures of paired MF lesion (A) and non-malignant skin (B) tissue samples from each individual (Figure 1). In this semi-quantitative array analysis, the transcription patterns of lesion samples were generally similar to those of non-malignant skin, in both MF and psoriasis patients, and showed only gradual differences in signal intensities. However, with the exception of patient 2 showing a HERV-H signal in non-malignant tissue but not in the lesion, a tendency towards increased HERV transcript levels in lesional tissue was observed at an individual level. The foremost group, HERV-W, appeared to be upregulated in skin lesions of MF patients compared to non-malignant skin. In addition, HERV-K (HML-6) and HERV-R showed some minor differences in transcript levels between non-malignant and lesion skin samples in both, MF and psoriasis patients (Figure 1). In contrast, low-level transcription of HERV-K (HML-2), and HML-3 was detected in lesion and/or non-malignant samples of a few MF patients, but not in any of the psoriasis patients.

### Significantly increased HERV-W transcript levels in mycosis fungoides

**Figure 2:**
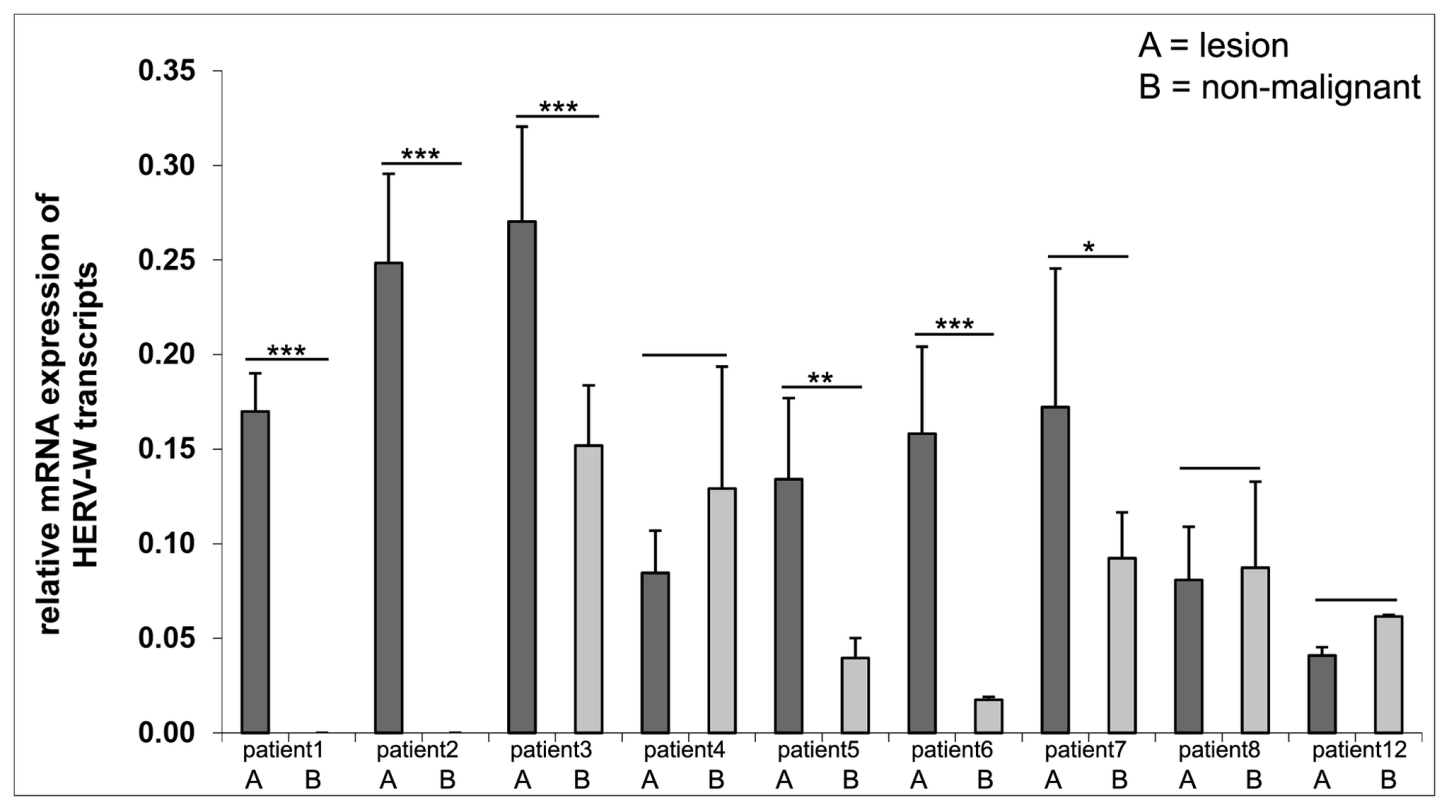
Relative quantification of specific HERV-W transcript levels. Lesion (A) and non-malignant (B) cDNAs from each patient (patients 1-8, 12, representing stages III, IA, IVA, fMF, fMF, IB, IA, IB, IB, in respective order) were amplified using HERV-W specific primers (targeting a region within the *pol* gene) and normalized with housekeeping gene RNA polymerase II. Lesion samples are depicted by dark grey bars, non-malignant intact skin samples by light gray bars. No data in patient1B and patient 2B means there was no detectable transcription. Relative mRNA expression levels are given as mean ±SD. Statistical significance is presented as *p<0.05, **p>0.02, and ***p<0.01. Concordance between the microarray and qRT-PCR data is not complete due to the differences in primer specificities (see Methods). fMF= folliculotrophic MF.

We next confirmed the differential HERV-W transcript levels identified with the semi-quantitative RetroArray analysis by quantitative RT-PCR on paired tissue samples of a representative set of patients. Of note, since the primers used in the RetroArray, amplify nearly all retroviral *pol* sequences (and since the amplification product would be too small), new primers were designed for the qRT-PCR in such a way that one primer matched the capture probe sequences spotted on the microarray, whereas the second primer was located 70 bp downstream of the first primer (see Methods). With qRT-PCR, the level of HERV-W transcripts was found to be significantly increased in 6 out of 9 (67%) MF skin lesions studied (representing disease stages IA-IVA and one folliculotropic MF, Figure 2).

### Identification of transcribed HERV-W loci

To identify active HERV-W loci, we next cloned the HERV-W specific RT-PCR products, sequenced randomly selected cDNA clones and assigned the cDNA sequences to HERV-W loci based on characteristic sequence differences between the various HERV-W *env* genes as described before [59]. With this method, the cloning frequency of cDNAs, i.e. transcripts from a HERV-W locus, roughly correlates with the transcript level of that specific HERV-W locus relative to other transcribed HERV-W loci.

**Figure 3:**
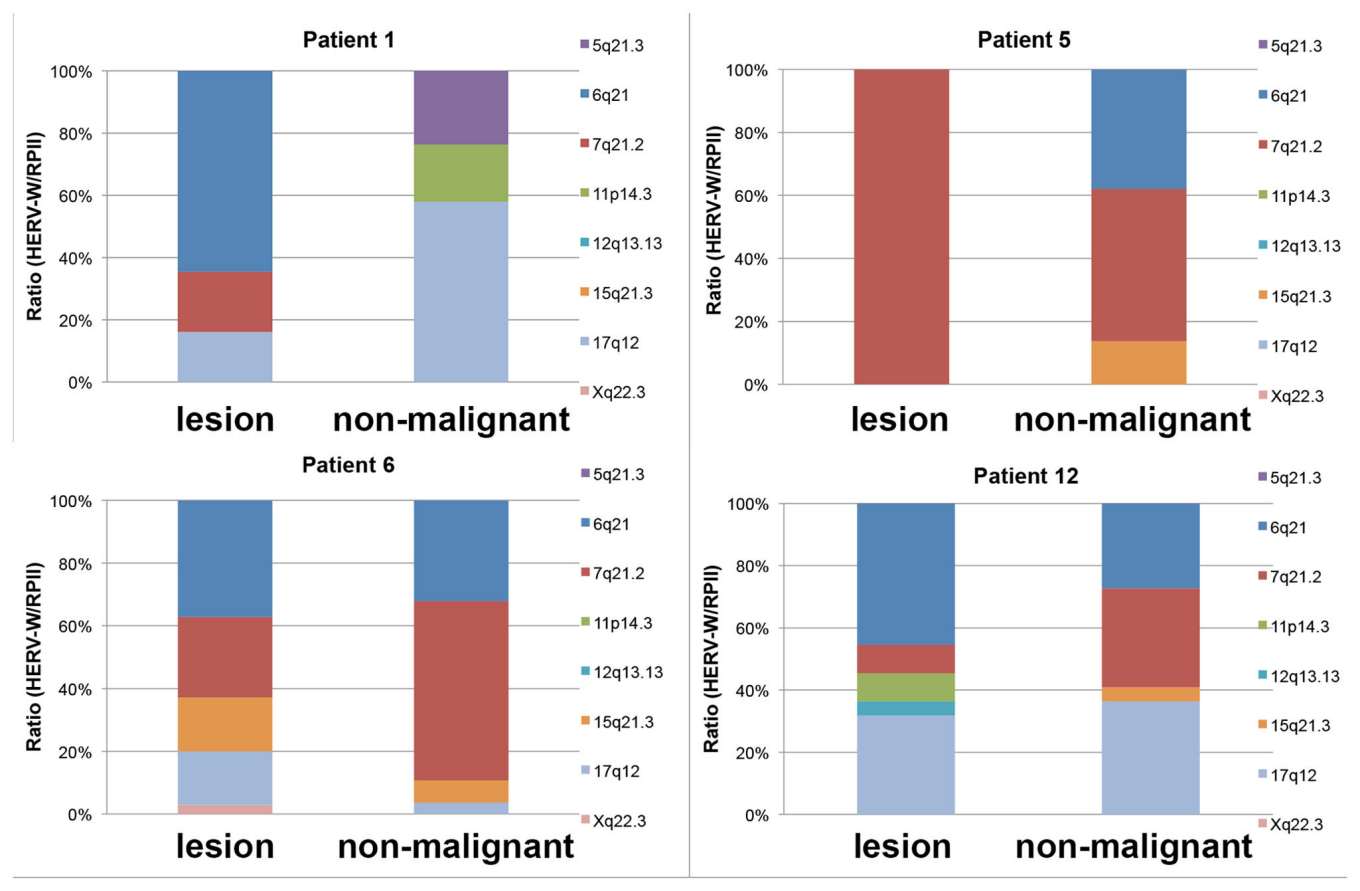
Transcriptional activity of HERV-W proviral loci in lesion and non-malignant skin tissue with MF. HERV-W transcripts were amplified using HERV-W *env* derived primers, cloned, sequenced and assigned to proviral loci as described previously [59]. For each proviral locus the relative cloning frequency is given as % of the total number of analyzed clones per skin tissue. HERV-W loci 6q21 and 7q21, 2 showed the highest relative cloning frequency, thus relative transcript levels in MF-lesions.

We sequenced between 22 and 38 (average: 29) cDNA clones from the MF lesion and non-malignant tissues of four patients (from which sufficient amount of cDNA was available). By so doing, we identified eight different HERV-W loci on chromosomes 5q21.3, 6q21, 7q21.2, 11p14.3, 12q13.13, 15q21.3, 17q12, and Xq22.3 as transcribed in at least one of the samples (Figure 3 and Table S2). Though conditions for amplification of transcripts derived from different HERV-W loci were the same, the number and the identity of transcribed HERV-W loci differed markedly between lesion and non-malignant tissue samples (Figure 3, Table S2). The HERV-W locus on chromosome 6q21 was cloned as cDNA most often, and transcribed at a higher level in MF lesion tissue than in the non-malignant tissue samples. HERV locus 5q21.3 was transcribed in non-lesional skin only while 17q12 was transcribed in both lesional and non-lesional tissue. Interestingly, in the MF lesional sample of patient 5, HERV-W transcripts appeared to be derived predominantly or exclusively (28/28 cDNA clones) from the 7q21.2 / *ERVWE1* locus. In summary, the highest, total numbers of cDNAs found in MF skin lesions and outnumbering the corresponding number in non-lesional skin were assigned to HERV-W loci 6q21 and in 7q21.2 (Figure 3, Table S2).

Five of these loci had been identified as transcribed in a recent study (see Table S1 and [59,61]). Thus, elevated HERV-W transcript levels detected by qRT-PCR in MF lesions appear to be due to up regulation of specific HERV-W loci, such as those located in 6q21 or 7q21.2. As for coding capacity of transcribed HERV-W loci, all loci but two display several frameshifts and stop mutations within the coding region for the Env protein. Therefore, protein production from those loci seems less likely. Importantly, the HERV-W locus in 7q21.2, *ERVWE1*, encodes a functional Env protein (Syncytin-1) that is crucially involved in placental morphogenesis [54-56]. Coding capacity of the transcribed *ERVWE2* locus in Xq22.3 has been addressed recently [62].

### ERVWE1 mRNA is present in mycosis fungoides skin lesions but not in benign lymphocyte infiltrates of the skin

**Figure 4:**
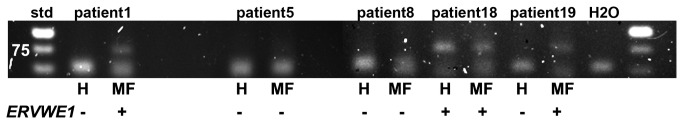
Relative expression of *ERVWE1* mRNA in MF skin tissue. *ERVWE1* expression was detected in 3/7 MF lesions analysed by Taqman qPCR. Patients 3 and 7 were also analysed, but the MF lesions showed no *ERVWE1* expression (Figure S1). Patients 7 and 18 showed expression also in the clinically healthy (H), non-lesional skin samples (the data of patient 7 in Figure S1). *GAPDH* served as internal standard and was expressed in every sample (see Methods for more details). MF patients 18 and 19 were collected afterwards and were therefore not included in other experiments. See detailed amplification curves in Figure S1.

Because a portion of the HERV-W transcripts were shown to be derived from the 7q21.2/*ERVWE1* locus, we used confirmatory Taqman qPCR to analyze the expression of *ERVWE1* mRNA in the MF lesions and also, for comparison, in five Lichen ruber planus skin samples (representing benign skin-homing T cell infiltrates). *ERVWE1* expression was detectable in 3/7 (43%) MF lesions analysed (Figure 4). The crossing point (CP) –values of *ERVWE1* ranged from 34 to 37 (see amplification curves as supportive information in Figure S1). *GAPDH* served as internal standard and was expressed in every sample (CP-values ranging from 19 to 23). The standard deviation within a sample pair (H, clinically non-lesional, i.e. healthy-looking skin vs. MF lesion) was less than 0.5 on average. Samples of the MF lesions of patients 3 and 7 were also analysed, but no expression was detected (Figure S1). On the contrary, patient 18 showed expression in both samples and patient 7 only in the non-lesional skin (Figure S1). Both of these patients had abundant numbers of MF patches and plaques over their skin at the time of biopsy, so although clinically healthy-looking, the skin may have been diseased. Most importantly, no *ERVWE1* mRNA expression was found in any of the five Lichen ruber planus samples studied (Figure S1).

### Syncytin-1 protein is expressed in mycosis fungoides skin lesions but not in lichen ruber planus

**Figure 5:**
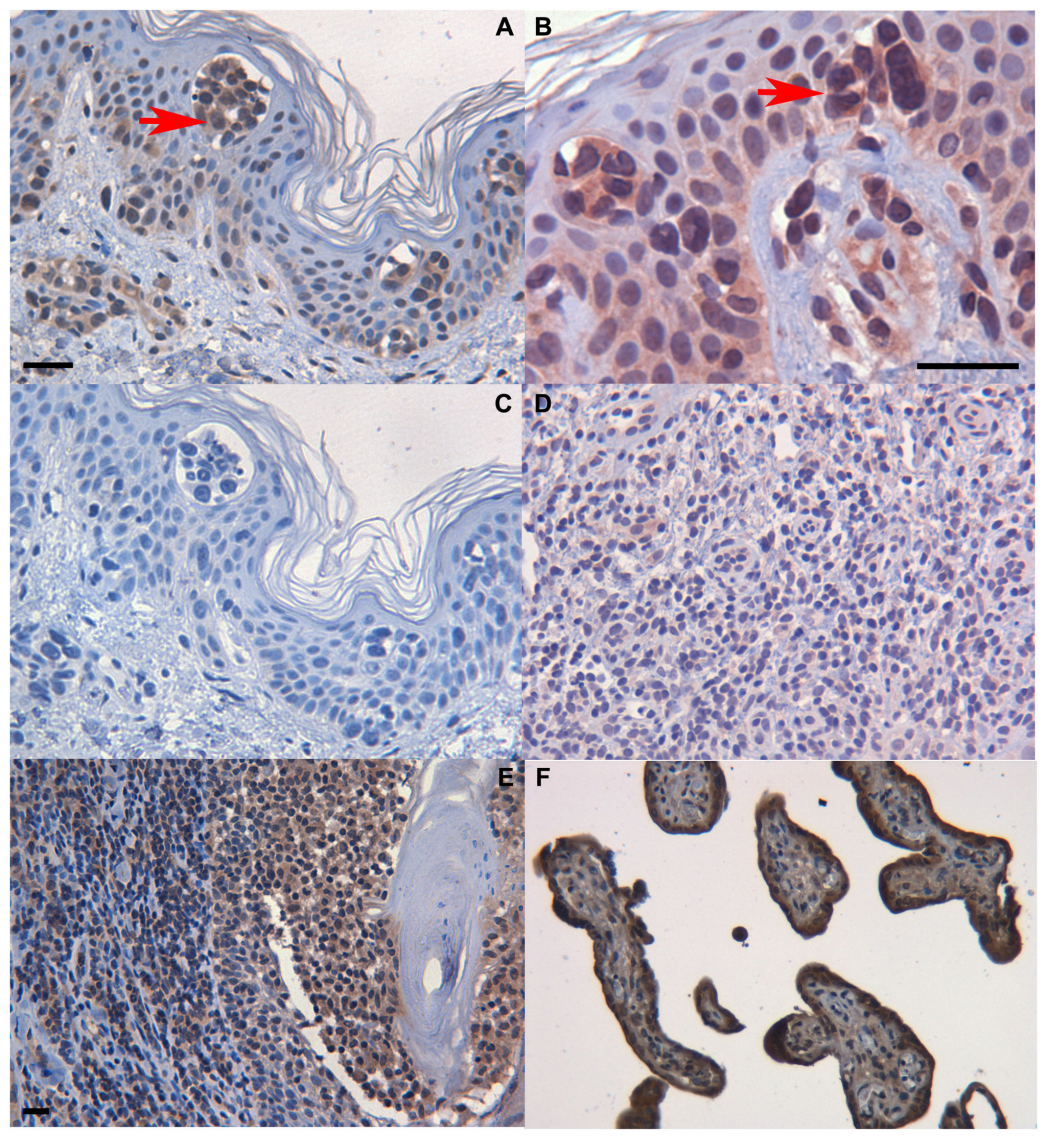
Syncytin-1 protein expression is found in MF skin lesions but not in lichen planus. A) Morphologically malignant lymphocytes within Pautrier’s microabscesses (invading the epidermis) stained positive (red arrow) for Syncytin-1 in immunohistochemistry, DAB 40x B) same sample as in a) but with NovaRED as a chromogen, 100x, C) same sample as in A) without primary antibody, DAB 40x, D) Lichen ruber planus sample with no Syncytin-1 expression albeit abundant numbers of inflammatory T cells, NovaRED 20x, E) in folliculotropic MF, Syncytin-1-positive lymphocytes protruding in the hair follicle, 20x and F) human placenta as a positive control, 20x. Scale bar is 20µm.

Since HERV-W transcripts derived from the 7q21.2/*ERVWE1* locus were frequently up regulated in the MF lesion tissue (Figures 3-4, S1), we next sought for possible expression of *ERVWE1* encoded Syncytin-1 (Env) protein. For this, we used monoclonal antibody 1F11B10, shown to recognize the surface-associated (SU) subunit of Syncytin-1, and immunohistochemistry (IHC) of an extended series of formalin-fixed, paraffin-embedded (FFPE)-skin biopsies, including also five samples of lichen ruber planus, an inflammatory dermatosis with abundant infiltrate of T lymphocytes. IHC revealed clear expression of Syncytin-1 in a varying number of lymphocytes in the sub-epidermal infiltrates or in individual lymphocytes invading the epidermis in 15/30 (50%) of the investigated MF cases (n=30, Figure 5, details in Methods). Typically, most abundant Syncytin-1 expression was seen in morphologically malignant lymphocyte clusters within the characteristic Pautrier’s microabscesses in the epidermis (Figure 5A-B). Syncytin-1 protein expression was detected in all but one (patient 5) of those MF skin samples from which active HERV loci had been cloned (Figure 3). No Syncytin-1 expression was detected in any of the lichen ruber planus samples (Figure 5D).

Additionally, patients 1, 7, 8, and 12 were available for serum sampling and their sera were examined for HERV-Wenv auto-antibodies using HERV recombinant ERVWE1 protein as antigen in ELISA assay. Serum from patient 12 showed antibody reactivity against this protein at a detectable level (OD value 0.8-1.0 with serum dilution 1:250, data not shown). Patient 12 was a male - thus not naturally sensitized to Syncytin-1 from placental expression of Syncytin-1 during pregnancy - and the only study subject without specific treatment during the preceding year and with relapsing skin lesions in three restricted skin regions.

## Discussion

This is the very first study to examine the transcription of HERVs in MF, the most common form of CTCL. Also, this is the first one to show expression of a HERV-W-encoded protein, Syncytin-1, in the CTCL lymphocytes. Our comprehensive microarray-based analysis identified a characteristic skin-specific HERV transcription profile and, in addition, certain degree of inter-individual variability in the HERV transcription patterns, as shown in previous studies on other human tissues (Table S1 and [39,60,63]). These observations jointly emphasize the importance of including paired samples of both malignant (lesion) and non-malignant (intact, healthy) tissue to a study. Since MF is a malignancy of sessile, non-recirculating skin-resident T-cells [3,64], and since there are no reliable methods to selectively isolate the malignant T-cells (and in sufficient numbers), relevant results could be obtained only by studying skin samples directly. Of note, even normal skin used in parallel, is known to contain resident effector memory T-cells. The reason for also including paired skin samples of psoriatic patients in our analyses was based on the recent report on the transcription of three HERV groups in psoriatic lesions, namely HERV-W, HERV-K and HERV-E, and partially characterized a new endogenous retroviral variant, related to the ERV-9⁄HERV-W group [48]. We confirmed all these HERV groups to be part of a constitutively transcribed, skin-specific HERV transcription profile. HERV-W was among the most strongly transcribed HERV groups in MF lesions (compared to the corresponding non-malignant skin samples). Our identification of the specific transcribed HERV-W elements yielded eight HERV-W loci on eight different chromosomes. Transcripts that could be ascribed to the HERV-W related, MS-associated retrovirus MSRV were not detected.

HERV-W is a multicopy human endogenous retrovirus group [65] that consists of about 1300 loci (including solo LTR, complete and partial loci [66]). In our study, HERV-W loci on chromosomes 6q21 and 7q21.2 were identified as being transcribed at higher levels in MF lesions (Table S2, Figure 3). Both loci have also been found transcribed in relation to neuroimmune diseases like MS in other studies [59,61,67]. Interestingly, frequent chromosomal alterations in CTCL also include chromosomes 6, 7, and 17. Recurrent breakpoints or deletions have been detected in e.g. 6q22-q25 and chromosomal gains in chromosome 7 [7-10,46,68]. Importantly, transformed MF tumor cells are characterized by gains of 7q21 and 17q12 among others [11]. Also, among patients with chronic lymphocytic leukemia (CLL) or with CTCL, the second most frequent chromosomal abnormalities were rearrangements involving 6q21-23 [69].

Syncytin-1 is a retroviral protein encoded by the HERV-W locus on chromosome 7q21.2 (official gene name: *ERVWE1*) [70]. It is a key player in human placental development, especially during fusion of cytotrophoblasts [54-56]. Cytotrophoblasts fuse to generate multinuclear syncytiotrophoblasts that function as pregnancy hormone factory [71,72]. Besides these beneficial physiological functions, Syncytin-1 is also capable of inducing neuroinflammation [44,73]. Fusogenic properties of Syncytin-1 outside the placenta are not well understood. Expression of Syncytin-1 outside the placenta has been reported, capable of activating a pro-inflammatory and autoimmune cascade through receptors CD14 and TLR4 (Toll-Like Receptor 4) on antigen-presenting cells, and also of triggering dysregulation of T-lymphocytes in multiple sclerosis (MS) [74]. *ERVWE1*-encoded Syncytin-1 protein expressed in MS is currently considered as an important therapeutic target. HERV-W elements appear associated with MS but it remains unclear whether they are triggers or markers.

Interestingly, we found most prominent Syncytin-1 expression in the morphologically malignant lymphocytes within the Pautrier´s microabscesses (Figure 5A-B) and also within the lymphocyte infiltrate invading hair follicles in folliculotropic MF (Figure 5E). The mechanism for the unique epidermotropism of malignant lymphocytes in MF has not been elucidated completely, although certain skin adressins like integrins, CLA and CCR4 play a role in general [64,75]. Our findings warrant further functional and prospective follow-up studies on the role of Syncytin-1 in the process of CTCL development.

## Conclusions

We report, for the first time, the transcription of HERVs in MF, the most common form of CTCL. However, it is still unclear, whether the elevated expression of HERV-W transcripts is due to malignant transformation, immune activation, or even physiological functions. Nevertheless, we also show the expression of a HERV-W-encoded protein, Syncytin-1, in skin-resident (i.e. skin-homing, non-recirculating) MF lymphocytes but not in non-malignant, skin-homing or skin-resident lymphocytes. Our study offers a new perspective into the pathogenesis of CTCL and it is tempting to speculate that Syncytin-1 could act as a proinflammatory stimulus in MF microenvironment in a similar fashion as in MS [43,44,76]. Still, we cannot totally exclude a possibility that HERV expression may only be a consequence of an inflammatory context although we did not detect any Syncytin-1 protein within the lymphocytic inflammatory infiltrates of lichen ruber planus. As a conclusion, we suggest that HERVs up regulated in various subsets of patients may represent promising candidates for future prospective studies identifying potential clinical correlates in CTCL and even novel therapeutic targets.

## Methods

### Ethics Statement

The study was approved by the Medical Ethical Review Board of Helsinki University Central Hospital and all patients provided a written informed consent. The investigation has been conducted according to the principles expressed in the Declaration of Helsinki.

### Patient samples and sample preparations

Surgical biopsies of both lesional and clinically healthy, non-lesional (i.e. non-malignant) skin were obtained from 12 patients with the common MF subtype of CTCL (10 with stage IA-IVA disease and two with folliculotropic MF) and five psoriasis patients, treated at the Skin and Allergy Hospital, Helsinki University Central Hospital (HUCH, Finland). The study was approved by the Medical Ethical Review Board of Helsinki University Central Hospital and all patients provided a written informed consent. None of the patients was infected with HIV-1 or with HTLV-I as determined with routine serology (www.huslab.fi). A total of 34 tissue samples were embedded in RNA stabilization reagent (Qiagen) immediately after surgery, immersed over night at +4°C and stored at -20°C or -70°C. RNA was extracted using RNeasy Mini Kit (Qiagen) and RNA was stored at -20°C or -70°C. Total RNA was incubated with 1 µl DNaseI (10 U/µl, recombinant, RNase-free, Roche Diagnostics, Mannheim, Germany) to remove genomic DNA. RNA quantity was measured by NanoDrop ND1000 (PeqLab Biotechnologie GmbH, Erlangen, Germany). About 25 ng of each RNA preparation was tested for DNA contamination by control PCR with mixed oligonucleotide primers (MOP) [37]. Only RNA preparations negative for PCR products in 3% agarose gel electrophoresis were used for subsequent reverse transcription with RevertAid First Strand cDNA Synthesis Kit (Fermentas, Helsinki, Finland) and for all consecutive RetroArray and qRT-PCR experiments. In all PCR experiments, negative controls were performed to exclude false positive signals derived from DNA contaminations.

For the additional experiments, we were able to collect another set of skin tissue samples (both lesion and clinically healthy) from five of the original MF patients with persistent disease (patients 1, 3, 5, 7, and 8) and from two recent MF patients (18 and 19). The RNA was extracted as described above. Additionally, RNA was also extracted from five formalin fixed and paraffin-embedded (FFPE) samples of lichen ruber planus using automated QIASymphony SP/AS instrument (Qiagen) which is designed and optimized especially for the purification of sufficient quality RNA from FFPE material. The reverse transcription into cDNA was performed using SuperScript®-VILO cDNA Synthesis kit (11754-050, Invitrogen). Moreover, additional 26 randomly selected, archival MF lesion and five lichen ruber planus FFPE samples (the same samples were used for RNA extraction, see above) were collected for Syncytin-1 IHC analysis.

### Retrovirus-specific Microarray (RetroArray)

The RetroArray consists of 48 representative HERV RT-derived sequences from 20 major HERV groups. For design of these capture probes, databases were screened for RT-related sequences, which were then classified according to the current nomenclature and further subgrouped with respect to their degree of nucleotide similarity. Representative members of each subgroup were selected with special emphasis on full-length proviral genomes and retroviral sequences that have been associated previously in literature with any biological activities and/or human diseases (see Table S1 in [77]). Mixed oligonucleotide primer (MOP) sequences were derived from two highly conserved amino acid motifs commonly found in all retroviral reverse transcriptase genes. Discrimination between different HERV subgroups was achieved by internal sequences of the amplification product. The internal sequences are specific for each HERV subgroup and bind to oligonucleotides (capture probes) spotted on the chip [37].

RetroArray hybridization probe synthesis, labelling by MOP PCR as well as printing, blocking, hybridization, and postprocessing of retrovirus-specific microarrays were performed as described previously [37,78,79]. Hybridized microarrays representing triplicates of the same array on the same chip were scanned using an Affymetrix GMS-418 scanner (laser power setting, 100%; gain setting, 50%), and the resulting images (16-bit TIFF files) were subjected to qualitative analysis using ImaGene 4.0 software (BioDiscovery, Inc., Los Angeles, USA). Exclusively arrays showing reproducible hybridization patterns in triplicate subarrays were further evaluated. Densitometric data were used for discrimination of positive signals from background.

As described in the study of [60], an arbitrary cut-off value corresponding to twofold background intensity values of the respective chip was used to discriminate between positive and negative signals. This proved to be in good agreement with the optical appearance of raw images when observed on a colour-calibrated monitor in a darkened room. To account for the influence of mRNA quality, HERV signals were compared to RNA levels of the gene HPRT, a housekeeping gene showing the most consistent transcript levels in urothelial tissues. Since RetroArray is in principle a qualitative method due to some design limitations (i.e. use of degenerate primers) as discussed in extenso [77,79,80], HERV groups showing signals above the mentioned cut-off value were evaluated as active regardless of their signal strength. It should be noted that each positive RetroArray signal may represent multiple HERV loci of one subgroup of multicopy HERV elements with sufficient sequence similarity to preclude identification of individual HERV loci. The original array data is available upon request from the authors.

### Relative quantification of HERV-W transcripts

Quantitative real-time PCR was performed with the Roche LightCycler 1.5 System, using LightCycler® 480 DNA SYBR Green I Master Mix according to the manufacturer’s instructions (Roche, Mannheim, Germany). For amplification of *pol* (reverse transcriptase domain) sequences, specific primers for group HERV-W (forward primer: TGAGTCAATTCTCAT-ACCTG, reverse primer: AGTTAAGAGTTCTTGGGTGG) were used [37]. The primers were designed in such a way that one primer matches the capture probe sequences used for the microarray experiments whereas the second primer is located 70 bp downstream of the first primer. Three independent replicates of amplifications were performed using a 10 minutes denaturation step at 95°C, followed by 40 cycles of 10 sec at 95°C, 5 sec at 60 °C, and 10 sec at 72°C. RNA-Polymerase II (RPII) transcripts were analyzed as internal standard, using primers given in [81]. To confirm specific amplification PCR products were further analyzed by melting curve analysis and by agarose gel electrophoresis (data not shown). The relative expression ratio was calculated as described [82] and statistical significance was measured using Two-Way ANOVA as implemented in the SPSS (Statistical Package for the Social Sciences) statistics software package (Figure 2).

### Identification of transcribed HERV loci

HERV-W specific RT-PCR products (HERV-W-derived transcripts) from patients 1, 5, 6, and 12 were purified (NucleoSpin Extract II, Macherey-Nagel, Düren, Germany) and cloned into the pGEM T-Easy vector (Promega) and transformed into TOP10F bacterial cells. Plasmid DNA was isolated from insert-containing colonies according to the manufacturer’s protocol (NucleoSpin Plasmid, Macherey-Nagel, Düren, Germany). Sequences of cloned RT-PCR products were determined using an Applied Biosystems 3730x Capillary Sequencer and using vector-specific primers (SeqIt GmbH, Kaiserlautern, Germany). The quality of sequencing chromatograms was assessed and poor quality reads were excluded from subsequent analysis.

We assigned sequences of cloned cDNAs to HERV-W genomic loci by BLAT searching the hg18 human reference genome sequence at the UCSC Genome Browser, employing randomly distributed and therefore characteristic sequence differences between the various HERV-W loci that allow discrimination between single loci [38,59,63] (Table S2, Figure 3).

We note that relative quantitation by RetroArray and qRT-PCR and identification of transcribed HERV-W loci utilized different primer sets. The RetroArray capture probes and qRT-PCR primers were located within the HERV-W *pol* gene. Primers for identification of transcribed HERV-W loci are located within the HERV-W *env* gene [59]. Many HERV-W loci lack 5' portions of different length because they were formed by L1-mediated retrotransposition [83,84]. Primers for identification of transcribed HERV-W loci can therefore identify, in principle, more transcribed HERV-W loci than primers used for qRT-PCR.

### ERVWE1 mRNA expression

The expression of *ERVWE1* was further confirmed with Taqman qPCR. The cDNAs were amplified on Roche LightCycler 480 1.5 System using validated Taqman Assays (0,5x, *ERVW1*, Hs00205893_m1 74bp, 0,5x *GAPDH* 4310884E, 118pb Life Technologies) and iQ Supermix (170-8860, Bio-Rad). The size and the purity of the amplicon was checked with agarose gel electrophoresis (3% SeaKem® LE agarose, Rockland, ME, USA, 1xTBE). *GAPDH* served as internal standard.

### Immunohistochemistry

Syncytin-1 protein was detected in formalin-fixed paraffin-embedded (FFPE) skin tissue samples of patients 1, 5, 6, and 12, obtained during the same time period as the ones used for the original RNA extraction and before any treatments (see Figure 2 for MF stages and section *Patient samples and sample preparations.*). Additionally, archival FFPE skin biopsies of histologically confirmed MF (n=26) and Lichen ruber planus (n=5) were also stained. The biopsies were obtained from untreated MF stage I - III lesions except for two cases under Targretin + PUVA therapy or recent chemotherapy. Two of the samples represented folliculotropic MF. The Lichen ruber planus samples served as non-malignant reference samples with abundant numbers of inflammatory T cells. IHC was performed using mouse monoclonal anti-syncytin-1 antibody (1:30, 4.38mg/ml, 1F11B10) with VECTASTAIN Elite ABC Kit (Mouse IgG, Vector Laboratories, Peterborough, UK) according to the manufacturers` instructions. As an antigen retrieval method, the slides were boiled in 10 mM sodiumcitrate buffer-0.05% Tween20, pH 6.0 for 20 minutes. The primary antibody was incubated over night in refrigerator. DAB or NovaRED was used as chromogen.

### Detection of auto-antibodies against recombinant HERV-Wenv protein

ELISA (Enzyme-Linked Immunosorbent Assay) was carried out to evaluate the presence of auto-antibodies against HERV-Wenv proteins in MF patient sera. We were able to collect sera from 4/12 MF patients (1, 7, 8, and 12). We used HERV Partial Recombinant Protein as an antigen (ERVWE1 116a.a.-216a.a, NP_055405, H00030816-Q01, Novus Biologicals, UK). Wells were coated with antigen dilution (0.75ug/ml, 22.5ng/well), incubated over night in refrigerator and washed three times with PBS-Tween0.05%. Blocking was done using 2% BSA/PBS for two hours in a shaker at RT. Each serum was diluted (1:50, 1:250, and 1:1250 in 0.5% BSA/PBS) and incubated for two hours in a shaker at RT, whereafter the plate was washed four times with PBS-Tween0.05%, and a secondary antibody (1:10000, goat anti-human-IgG-HRP, 109-035-098 Jackson ImmunoResearch laboratories Inc, West Grove, PA, U.S.A) was added and incubated for one hour in a shaker at RT. After washing, the bound antibody was detected with 1-Step™ Ultra TMB-Elisa reagent (34028 Thermo scientific, Rockford, IL, U.S.A). Absorption was measured at 450 nm. Blank control samples lacked the antigen.

## Supporting Information

Figure S1 **Amplification curves of studied MF and lichen ruber planus samples.** A) ERVWE1 amplification in patient 1 (H-, MF+), B) no ERVWE1 amplification in patient 3, C) no ERVWE1 amplification in patient 5, D) ERVWE1 amplification in patient 7 (H+, MF-), E) no ERVWE1 amplification in patient 8, F) ERVWE1 amplification in patient 18 (H+, MF+), G) ERVWE1 amplification in patient 19 (H-, MF+), H) GAPDH amplification in all studied MF samples (red), also negative water controls are shown (green baseline), I) no ERVWE1 amplification in any of the Lichen ruber planus samples (n=5), and J) GAPDH (red) versus ERVWE1 (green) amplification of all studied Lichen ruber planus samples. Also negative water controls are presented (green baseline). H= clinically healthy, non-lesional, MF= MF lesion, + indicates amplification, - no amplification.
(PDF)

Table S1 **Comparison of HERV core transcription pattern in human tissues.**
(PDF)

Table S2 **Summary of assignment of lesion and non-malignant skin tissue derived cDNA sequences to specific HERV-W loci.**
(PDF)

## References

[1] Parker SR, Bethaney JV (2009) Cutaneous T cell lymphoma-mycosis fungoides and sezary syndrome: An update. G Ital Dermatol Venereol 144: 467-485. PubMed: 19755952. 19755952

[2] Kempf W, Sander CA (2010) Classification of cutaneous lymphomas - an update. Histopathology 56: 57-70. doi:10.1111/j.1365-2559.2009.03455.x. PubMed: 20055905. 20055905 10.1111/j.1365-2559.2009.03455.x

[3] Clark RA, Watanabe R, Teague JE, Schlapbach C, Tawa MC et al. (2012) Skin effector memory T cells do not recirculate and provide immune protection in alemtuzumab-treated CTCL patients. Sci Transl Med 4: 117ra7- 10.1126/scitranslmed.3003008 PMC3373186 22261031

[4] Muche JM, Sterry W, Gellrich S, Rzany B, Audring H et al. (2003) Peripheral blood T-cell clonality in mycosis fungoides and nonlymphoma controls. Diagn Mol Pathol 12: 142-150.- doi:10.1097/00019606-200309000-00005. PubMed: 12960696. 12960696 10.1097/00019606-200309000-00005

[5] Karenko L, Kähkönen M, Hyytinen ER, Lindlof M, Ranki A (1999) Notable losses at specific regions of chromosomes 10q and 13q in the sezary syndrome detected by comparative genomic hybridization. J Invest Dermatol 112: 392-395. doi:10.1038/sj.jid.5600444. PubMed: 10084322. 10084322 10.1038/sj.jid.5600444

[6] Mao X, Lillington DM, Czepulkowski B, Russell-Jones R, Young BD et al. (2003) Molecular cytogenetic characterization of sezary syndrome. Genes Chromosomes Cancer 36: 250-260. doi:10.1002/gcc.10152. PubMed: 12557225. 12557225 10.1002/gcc.10152

[7] Espinet B, Salido M, Pujol RM, Florensa L, Gallardo F et al. (2004) Genetic characterization of sezary’s syndrome by conventional cytogenetics and cross-species color banding fluorescent in situhybridization. Haematologica 89: 165-173. PubMed: 15003891. 15003891

[8] Batista DA, Vonderheid EC, Hawkins A, Morsberger L, Long P et al. (2006) Multicolor fluorescence in situ hybridization (SKY) in mycosis fungoides and sezary syndrome: Search for recurrent chromosome abnormalities. Genes Chromosomes Cancer 45: 383-391. doi:10.1002/gcc.20302. PubMed: 16382449. 16382449 10.1002/gcc.20302

[9] Karenko L, Hahtola S, Päivinen S, Karhu R, Syrjä S et al. (2005) Primary cutaneous T-cell lymphomas show a deletion or translocation affecting NAV3, the human UNC-53 homologue. Cancer Res 65: 8101-8110. doi:10.1158/0008-5472.CAN-04-0366. PubMed: 16166283. 16166283 10.1158/0008-5472.CAN-04-0366

[10] Vermeer MH, van Doorn R, Dijkman R, Mao X, Whittaker S et al. (2008) Novel and highly recurrent chromosomal alterations in sezary syndrome. Cancer Res 68: 2689-2698. doi:10.1158/0008-5472.CAN-07-6398. PubMed: 18413736. 18413736 10.1158/0008-5472.CAN-07-6398

[11] Laharanne E, Chevret E, Idrissi Y, Gentil C, Longy M et al. (2010) CDKN2A-CDKN2B deletion defines an aggressive subset of cutaneous T-cell lymphoma. Mod Pathol 23: 547-558. doi:10.1038/modpathol.2009.196. PubMed: 20118908. 20118908 10.1038/modpathol.2009.196

[12] Lin WM, Girardi M (2010) More or less: Copy number alterations in mycosis fungoides. J Invest Dermatol 130: 926-928. doi:10.1038/jid.2009.370. PubMed: 20231832. 20231832 10.1038/jid.2009.370

[13] Tan RS, Butterworth CM, McLaughlin H, Malka S, Samman PD (1974) Mycosis fungoides--a disease of antigen persistence. Br J Dermatol 91: 607-616. doi:10.1111/j.1365-2133.1974.tb12449.x. PubMed: 4281316. 4281316 10.1111/j.1365-2133.1974.tb12449.x

[14] Beyer M, Möbs M, Humme D, Sterry W (2011) Pathogenesis of mycosis fungoides. J Dtsch Dermatol Ges 9: 594-598. doi:10.1111/j.1610-0387.2011.07635.x. PubMed: 21371258. 21371258 10.1111/j.1610-0387.2011.07635.x

[15] Saxinger WC, Wantzin GL, Thomsen K, Hoh M, Gallo RC (1985) Occurrence of HTLV-I antibodies in danish patients with cutaneous T-cell lymphoma. Scand J Haematol 34: 455-462. PubMed: 2990023. 2990023 10.1111/j.1600-0609.1985.tb00777.x

[16] Ranki A, Niemi KM, Nieminen P, Krohn K (1990) Antibodies against retroviral core proteins in relation to disease outcome in patients with mycosis fungoides. Arch Dermatol Res 282: 532-538. doi:10.1007/BF00371949. PubMed: 2082836. 2082836 10.1007/BF00371949

[17] Pancake BA, Zucker-Franklin D, Coutavas EE (1995) The cutaneous T cell lymphoma, mycosis fungoides, is a human T cell lymphotropic virus-associated disease. A study of 50 patients. J Clin Invest 95: 547-554. doi:10.1172/JCI117697. PubMed: 7860737. 7860737 10.1172/JCI117697 PMC295510

[18] Bazarbachi A, Soriano V, Pawson R, Vallejo A, Moudgil T et al. (1997) Mycosis fungoides and sezary syndrome are not associated with HTLV-I infection: An international study. Br J Haematol 98: 927-933. doi:10.1046/j.1365-2141.1997.3213138.x. PubMed: 9326191. 9326191 10.1046/j.1365-2141.1997.3213138.x

[19] Kikuchi A, Nishikawa T, Ikeda Y, Yamaguchi K (1997) Absence of human T-lymphotropic virus type I in japanese patients with cutaneous T-cell lymphoma. Blood 89: 1529-1532. PubMed: 9057633. 9057633

[20] Wood GS, Schaffer JM, Boni R, Dummer R, Burg G et al. (1997) No evidence of HTLV-I proviral integration in lymphoproliferative disorders associated with cutaneous T-cell lymphoma. Am J Pathol 150: 667-673. PubMed: 9033279. 9033279 PMC1858284

[21] Zendri E, Pilotti E, Perez M, Turci M, Pinelli S et al. (2008) The HTLV tax-like sequences in cutaneous T-cell lymphoma patients. J Invest Dermatol 128: 489-492. doi:10.1038/sj.jid.5701034. PubMed: 17762859. 17762859 10.1038/sj.jid.5701034

[22] Dereure O, Cheval J, Du Thanh A, Pariente K, Sauvage V et al. (2013) No evidence for viral sequences in mycosis fungoides and sezary syndrome skin lesions: A high-throughput sequencing approach. J Invest Dermatol 133: 853-855. doi:10.1038/jid.2012.371. PubMed: 23096719. 23096719 10.1038/jid.2012.371

[23] Greenwood AD, Stengel A, Erfle V, Seifarth W, Leib-Mösch C (2005) The distribution of pol containing human endogenous retroviruses in non-human primates. Virology 334: 203-213. doi:10.1016/j.virol.2005.01.045. PubMed: 15780870. 15780870 10.1016/j.virol.2005.01.045

[24] Stoye JP (2012) Studies of endogenous retroviruses reveal a continuing evolutionary saga. Nat Rev Microbiol 10: 395-406. PubMed: 22565131. 22565131 10.1038/nrmicro2783

[25] Ruprecht K, Mayer J, Sauter M, Roemer K, Mueller-Lantzsch N (2008) Endogenous retroviruses and cancer. Cell Mol Life Sci 65: 3366-3382. doi:10.1007/s00018-008-8496-1. PubMed: 18818873. 18818873 10.1007/s00018-008-8496-1 PMC11131668

[26] Kozeretska IA, Demydov SV, Ostapchenko LI (2011) Mobile genetic elements and cancer. from mutations to gene therapy. Exp Oncol 33: 198-205. PubMed: 22217707. 22217707

[27] Mullins CS, Linnebacher M (2012) Human endogenous retroviruses and cancer: Causality and therapeutic possibilities. World J Gastroenterol 18: 6027-6035. doi:10.3748/wjg.v18.i42.6027. PubMed: 23155332. 23155332 10.3748/wjg.v18.i42.6027 PMC3496880

[28] Belshaw R, Katzourakis A, Paces J, Burt A, Tristem M (2005) High copy number in human endogenous retrovirus families is associated with copying mechanisms in addition to reinfection. Mol Biol Evol 22: 814-817. doi:10.1093/molbev/msi088. PubMed: 15659556. 15659556 10.1093/molbev/msi088

[29] Katzourakis A, Rambaut A, Pybus OG (2005) The evolutionary dynamics of endogenous retroviruses. Trends Microbiol 13: 463-468. doi:10.1016/j.tim.2005.08.004. PubMed: 16109487. 16109487 10.1016/j.tim.2005.08.004

[30] Hohenadl C, Germaier H, Walchner M, Hagenhofer M, Herrmann M et al. (1999) Transcriptional activation of endogenous retroviral sequences in human epidermal keratinocytes by UVB irradiation. J Invest Dermatol 113: 587-594. doi:10.1046/j.1523-1747.1999.00728.x. PubMed: 10504445. 10504445 10.1046/j.1523-1747.1999.00728.x

[31] Zeilfelder U, Frank O, Sparacio S, Schön U, Bosch V et al. (2007) The potential of retroviral vectors to cotransfer human endogenous retroviruses (HERVs) from human packaging cell lines. Gene 390: 175-179. doi:10.1016/j.gene.2006.08.019. PubMed: 17045761. 17045761 10.1016/j.gene.2006.08.019

[32] Garrison KE, Jones RB, Meiklejohn DA, Anwar N, Ndhlovu LC et al. (2007) T cell responses to human endogenous retroviruses in HIV-1 infection. PLOS Pathog 3: e165. doi:10.1371/journal.ppat.0030165. 17997601 10.1371/journal.ppat.0030165 PMC2065876

[33] Ruprecht K, Obojes K, Wengel V, Gronen F, Kim KS et al. (2006) Regulation of human endogenous retrovirus W protein expression by herpes simplex virus type 1: Implications for multiple sclerosis. J Neurovirol 12: 65-71. doi:10.1080/13550280600614973. PubMed: 16595376. 16595376 10.1080/13550280600614973

[34] Weiss RA (2006) The discovery of endogenous retroviruses. Retrovirology 3: 67. doi:10.1186/1742-4690-3-S1-S67. PubMed: 17018135. 17018135 10.1186/1742-4690-3-67 PMC1617120

[35] Moyes D, Griffiths DJ, Venables PJ (2007) Insertional polymorphisms: A new lease of life for endogenous retroviruses in human disease. Trends Genet 23: 326-333. doi:10.1016/j.tig.2007.05.004. PubMed: 17524519. 17524519 10.1016/j.tig.2007.05.004

[36] Stauffer Y, Theiler G, Sperisen P, Lebedev Y, Jongeneel CV (2004) Digital expression profiles of human endogenous retroviral families in normal and cancerous tissues. Cancer Immun 4: 2 PubMed: 14871062. 14871062

[37] Seifarth W, Frank O, Zeilfelder U, Spiess B, Greenwood AD et al. (2005) Comprehensive analysis of human endogenous retrovirus transcriptional activity in human tissues with a retrovirus-specific microarray. J Virol 79: 341-352. doi:10.1128/JVI.79.1.341-352.2005. PubMed: 15596828. 15596828 10.1128/JVI.79.1.341-352.2005 PMC538696

[38] Flockerzi A, Ruggieri A, Frank O, Sauter M, Maldener E et al. (2008) Expression patterns of transcribed human endogenous retrovirus HERV-K(HML-2) loci in human tissues and the need for a HERV transcriptome project. BMC Genomics 9: 354. doi:10.1186/1471-2164-9-354. PubMed: 18664271. 18664271 10.1186/1471-2164-9-354 PMC2525661

[39] Haupt S, Tisdale M, Vincendeau M, Clements MA, Gauthier DT et al. (2011) Human endogenous retrovirus transcription profiles of the kidney and kidney-derived cell lines. J Gen Virol 92: 2356-2366. doi:10.1099/vir.0.031518-0. PubMed: 21697344. 21697344 10.1099/vir.0.031518-0

[40] Pichon JP, Bonnaud B, Cleuziat P, Mallet F (2006) Multiplex degenerate PCR coupled with an oligo sorbent array for human endogenous retrovirus expression profiling. Nucleic Acids Res 34: e46. doi:10.1093/nar/gkl086. PubMed: 16554552. 16554552 10.1093/nar/gkl086 PMC1409818

[41] Pérot P, Mugnier N, Montgiraud C, Gimenez J, Jaillard M et al. (2012) Microarray-based sketches of the HERV transcriptome landscape. PLOS ONE 7: e40194. doi:10.1371/journal.pone.0040194. PubMed: 22761958. 22761958 10.1371/journal.pone.0040194 PMC3386233

[42] Romanish MT, Cohen CJ, Mager DL (2010) Potential mechanisms of endogenous retroviral-mediated genomic instability in human cancer. Semin Cancer Biol 20: 246-253. doi:10.1016/j.semcancer.2010.05.005. PubMed: 20685251. 20685251 10.1016/j.semcancer.2010.05.005

[43] Brudek T, Christensen T, Hansen HJ, Petersen T, Møller-Larsen A (2008) Synergistic immune responses induced by endogenous retrovirus and herpesvirus antigens result in increased production of inflammatory cytokines in multiple sclerosis patients. Scand J Immunol 67: 295-303. doi:10.1111/j.1365-3083.2007.02067.x. PubMed: 18261041. 18261041 10.1111/j.1365-3083.2007.02067.x

[44] Antony JM, Ellestad KK, Hammond R, Imaizumi K, Mallet F et al. (2007) The human endogenous retrovirus envelope glycoprotein, syncytin-1, regulates neuroinflammation and its receptor expression in multiple sclerosis: A role for endoplasmic reticulum chaperones in astrocytes. J Immunol 179: 1210-1224. PubMed: 17617614. 17617614 10.4049/jimmunol.179.2.1210

[45] Johnston JB, Silva C, Holden J, Warren KG, Clark AW et al. (2001) Monocyte activation and differentiation augment human endogenous retrovirus expression: Implications for inflammatory brain diseases. Ann Neurol 50: 434-442. doi:10.1002/ana.1131. PubMed: 11601494. 11601494 10.1002/ana.1131

[46] Katsumata K, Ikeda H, Sato M, Ishizu A, Kawarada Y et al. (1999) Cytokine regulation of env gene expression of human endogenous retrovirus-R in human vascular endothelial cells. Clin Immunol 93: 75-80. doi:10.1006/clim.1999.4762. PubMed: 10497013. 10497013 10.1006/clim.1999.4762

[47] Mameli G, Astone V, Khalili K, Serra C, Sawaya BE et al. (2007) Regulation of the syncytin-1 promoter in human astrocytes by multiple sclerosis-related cytokines. Virology 362: 120-130. doi:10.1016/j.virol.2006.12.019. PubMed: 17258784. 17258784 10.1016/j.virol.2006.12.019

[48] Molès JP, Tesniere A, Guilhou JJ (2005) A new endogenous retroviral sequence is expressed in skin of patients with psoriasis. Br J Dermatol 153: 83-89. doi:10.1111/j.1365-2133.2005.06555.x. PubMed: 16029331. 16029331 10.1111/j.1365-2133.2005.06555.x

[49] van de Lagemaat LN, Landry JR, Mager DL, Medstrand P (2003) Transposable elements in mammals promote regulatory variation and diversification of genes with specialized functions. Trends Genet 19: 530-536. doi:10.1016/j.tig.2003.08.004. PubMed: 14550626. 14550626 10.1016/j.tig.2003.08.004

[50] Conley AB, Piriyapongsa J, Jordan IK (2008) Retroviral promoters in the human genome. Bioinformatics 24: 1563-1567. doi:10.1093/bioinformatics/btn243. PubMed: 18535086. 18535086 10.1093/bioinformatics/btn243

[51] Jern P, Coffin JM (2008) Effects of retroviruses on host genome function. Annu Rev Genet 42: 709-732. doi:10.1146/annurev.genet.42.110807.091501. PubMed: 18694346. 18694346 10.1146/annurev.genet.42.110807.091501

[52] Feschotte C, Gilbert C (2012) Endogenous viruses: Insights into viral evolution and impact on host biology. Nat Rev Genet 13: 283-296. doi:10.1038/nrg3199. PubMed: 22421730. 22421730 10.1038/nrg3199

[53] Rebollo R, Romanish MT, Mager DL (2012) Transposable elements: An abundant and natural source of regulatory sequences for host genes. Annu Rev Genet, 46: 21–42. PubMed: 22905872. 22905872 10.1146/annurev-genet-110711-155621

[54] Blond JL, Lavillette D, Cheynet V, Bouton O, Oriol G et al. (2000) An envelope glycoprotein of the human endogenous retrovirus HERV-W is expressed in the human placenta and fuses cells expressing the type D mammalian retrovirus receptor. J Virol 74: 3321-3329. doi:10.1128/JVI.74.7.3321-3329.2000. PubMed: 10708449. 10708449 10.1128/jvi.74.7.3321-3329.2000 PMC111833

[55] Mi S, Lee X, Li X, Veldman GM, Finnerty H et al. (2000) Syncytin is a captive retroviral envelope protein involved in human placental morphogenesis. Nature 403: 785-789. doi:10.1038/35001608. PubMed: 10693809. 10693809 10.1038/35001608

[56] Mallet F, Bouton O, Prudhomme S, Cheynet V, Oriol G et al. (2004) The endogenous retroviral locus ERVWE1 is a bona fide gene involved in hominoid placental physiology. Proc Natl Acad Sci U S A 101: 1731-1736. doi:10.1073/pnas.0305763101. PubMed: 14757826. 14757826 10.1073/pnas.0305763101 PMC341840

[57] Antony JM, van Marle G, Opii W, Butterfield DA, Mallet F et al. (2004) Human endogenous retrovirus glycoprotein-mediated induction of redox reactants causes oligodendrocyte death and demyelination. Nat Neurosci 7: 1088-1095. doi:10.1038/nn1319. PubMed: 15452578. 15452578 10.1038/nn1319

[58] Perron H, Garson JA, Bedin F, Beseme F, Paranhos-Baccala G et al. (1997) Molecular identification of a novel retrovirus repeatedly isolated from patients with multiple sclerosis. the collaborative research group on multiple sclerosis. Proc Natl Acad Sci U S A 94: 7583-7588. doi:10.1073/pnas.94.14.7583. PubMed: 9207135. 9207135 10.1073/pnas.94.14.7583 PMC23865

[59] Laufer G, Mayer J, Mueller BF, Mueller-Lantzsch N, Ruprecht K (2009) Analysis of transcribed human endogenous retrovirus W env loci clarifies the origin of multiple sclerosis-associated retrovirus env sequences. Retrovirology 6: 37. doi:10.1186/1742-4690-6-S2-P37. PubMed: 19368703. 19368703 10.1186/1742-4690-6-37 PMC2672075

[60] Frank O, Giehl M, Zheng C, Hehlmann R, Leib-Mösch C et al. (2005) Human endogenous retrovirus expression profiles in samples from brains of patients with schizophrenia and bipolar disorders. J Virol 79: 10890-10901. doi:10.1128/JVI.79.17.10890-10901.2005. PubMed: 16103141. 16103141 10.1128/JVI.79.17.10890-10901.2005 PMC1193590

[61] Mayer J, Blomberg J, Seal RL (2011) A revised nomenclature for transcribed human endogenous retroviral loci. Mob DNA 2: 7. doi:10.1186/1759-8753-2-7. PubMed: 21542922. 21542922 10.1186/1759-8753-2-7 PMC3113919

[62] Roebke C, Wahl S, Laufer G, Stadelmann C, Sauter M et al. (2010) An N-terminally truncated envelope protein encoded by a human endogenous retrovirus W locus on chromosome Xq22.3. Retrovirology 7: 69. doi:10.1186/1742-4690-7-69. PubMed: 20735848. 20735848 10.1186/1742-4690-7-69 PMC2936387

[63] Frank O, Verbeke C, Schwarz N, Mayer J, Fabarius A et al. (2008) Variable transcriptional activity of endogenous retroviruses in human breast cancer. J Virol 82: 1808-1818. doi:10.1128/JVI.02115-07. PubMed: 18077721. 18077721 10.1128/JVI.02115-07 PMC2258715

[64] Campbell JJ, Clark RA, Watanabe R, Kupper TS (2010) Sezary syndrome and mycosis fungoides arise from distinct T-cell subsets: A biologic rationale for their distinct clinical behaviors. Blood 116: 767-771. doi:10.1182/blood-2009-11-251926. PubMed: 20484084. 20484084 10.1182/blood-2009-11-251926 PMC2918332

[65] Blond JL, Besème F, Duret L, Bouton O, Bedin F et al. (1999) Molecular characterization and placental expression of HERV-W, a new human endogenous retrovirus family. J Virol 73: 1175-1185. PubMed: 9882319. 9882319 10.1128/jvi.73.2.1175-1185.1999 PMC103938

[66] Mager DL, Medstrand P (2003) Retroviral repeat sequences. In: Anonymous ENCYCLOPEDIA OF THE HUMAN GENOME 5: 57-63.

[67] Antony JM, DesLauriers AM, Bhat RK, Ellestad KK, Power C (2011) Human endogenous retroviruses and multiple sclerosis: Innocent bystanders or disease determinants? Biochim Biophys Acta 1812: 162–176. doi:10.1016/j.bbadis.2010.07.016. PubMed: 20696240. 20696240 10.1016/j.bbadis.2010.07.016 PMC7172332

[68] Fischer TC, Gellrich S, Muche JM, Sherev T, Audring H et al. (2004) Genomic aberrations and survival in cutaneous T cell lymphomas. J Invest Dermatol 122: 579-586. doi:10.1111/j.0022-202X.2004.22301.x. PubMed: 15086538. 15086538 10.1111/j.0022-202X.2004.22301.x

[69] Nowell PC, Vonderheid EC, Besa E, Hoxie JA, Moreau L et al. (1986) The most common chromosome change in 86 chronic B cell or T cell tumors: A 14q32 translocation. Cancer Genet Cytogenet 19: 219-227. doi:10.1016/0165-4608(86)90050-6. PubMed: 3484667. 3484667 10.1016/0165-4608(86)90050-6

[70] Cheynet V, Ruggieri A, Oriol G, Blond JL, Boson B et al. (2005) Synthesis, assembly, and processing of the env ERVWE1/syncytin human endogenous retroviral envelope. J Virol 79: 5585-5593. doi:10.1128/JVI.79.9.5585-5593.2005. PubMed: 15827173. 15827173 10.1128/JVI.79.9.5585-5593.2005 PMC1082723

[71] Frendo JL, Olivier D, Cheynet V, Blond JL, Bouton O et al. (2003) Direct involvement of HERV-W env glycoprotein in human trophoblast cell fusion and differentiation. Mol Cell Biol 23: 3566-3574. doi:10.1128/MCB.23.10.3566-3574.2003. PubMed: 12724415. 12724415 10.1128/MCB.23.10.3566-3574.2003 PMC164757

[72] Handwerger S (2010) New insights into the regulation of human cytotrophoblast cell differentiation. Mol Cell Endocrinol 323: 94-104. doi:10.1016/j.mce.2009.12.015. PubMed: 20036312. 20036312 10.1016/j.mce.2009.12.015 PMC2874088

[73] Bhat RK, Ellestad KK, Wheatley BM, Warren R, Holt RA et al. (2011) Age- and disease-dependent HERV-W envelope allelic variation in brain: Association with neuroimmune gene expression. PLOS ONE 6: e19176. doi:10.1371/journal.pone.0019176. PubMed: 21559469. 21559469 10.1371/journal.pone.0019176 PMC3084769

[74] Perron H, Bernard C, Bertrand JB, Lang AB, Popa I et al. (2009) Endogenous retroviral genes, herpesviruses and gender in multiple sclerosis. J Neurol Sci 286: 65-72. doi:10.1016/j.jns.2009.04.034. PubMed: 19447411. 19447411 10.1016/j.jns.2009.04.034

[75] Schechner JS, Edelson RL, McNiff JM, Heald PW, Pober JS (1999) Integrins alpha4beta7 and alphaEbeta7 are expressed on epidermotropic T cells in cutaneous T cell lymphoma and spongiotic dermatitis. Lab Invest 79: 601-607. PubMed: 10334571. 10334571

[76] Rolland A, Jouvin-Marche E, Viret C, Faure M, Perron H et al. (2006) The envelope protein of a human endogenous retrovirus-W family activates innate immunity through CD14/TLR4 and promotes Th1-like responses. J Immunol 176: 7636-7644. PubMed: 16751411. 16751411 10.4049/jimmunol.176.12.7636

[77] Gosenca D, Gabriel U, Steidler A, Mayer J, Diem O et al. (2012) HERV-E-mediated modulation of PLA2G4A transcription in urothelial carcinoma. PLOS ONE 7: e49341. doi:10.1371/journal.pone.0049341. PubMed: 23145155. 23145155 10.1371/journal.pone.0049341 PMC3492278

[78] Seifarth W, Spiess B, Zeilfelder U, Speth C, Hehlmann R et al. (2003) Assessment of retroviral activity using a universal retrovirus chip. J Virol Methods 112: 79-91. doi:10.1016/S0166-0934(03)00194-0. PubMed: 12951215. 12951215 10.1016/s0166-0934(03)00194-0

[79] Seifarth W, Frank O, Schreml J, Leib-Mösch C (2011) RetroArray – a comprehensive diagnostic DNA chip for rapid detection and identification of retroviruses, retroviral contaminants, and mistaken identity of cell lines. In: Duncan S Wiley P Encyclopedia of DNA research. Hauppauge, NY: Nova Science Publishers, Inc.

[80] Seifarth W, Krause U, Hohenadl C, Baust C, Hehlmann R et al. (2000) Rapid identification of all known retroviral reverse transcriptase sequences with a novel versatile detection assay. AIDS Res Hum Retrovir 16: 721-729. doi:10.1089/088922200308729. PubMed: 10826479. 10826479 10.1089/088922200308729

[81] Radonić A, Thulke S, Mackay IM, Landt O, Siegert W et al. (2004) Guideline to reference gene selection for quantitative real-time PCR. Biochem Biophys Res Commun 313: 856-862. doi:10.1016/j.bbrc.2003.11.177. PubMed: 14706621. 14706621 10.1016/j.bbrc.2003.11.177

[82] Pfaffl MW (2001) A new mathematical model for relative quantification in real-time RT-PCR. Nucleic Acids Res 29: e45. doi:10.1093/nar/29.9.e45. PubMed: 11328886. 11328886 10.1093/nar/29.9.e45 PMC55695

[83] Costas J (2002) Characterization of the intragenomic spread of the human endogenous retrovirus family HERV-W. Mol Biol Evol 19: 526-533. doi:10.1093/oxfordjournals.molbev.a004108. PubMed: 11919294. 11919294 10.1093/oxfordjournals.molbev.a004108

[84] Pavlícek A, Paces J, Elleder D, Hejnar J (2002) Processed pseudogenes of human endogenous retroviruses generated by LINEs: Their integration, stability, and distribution. Genome Res 12: 391-399. doi:10.1101/gr.216902. PubMed: 11875026. 11875026 10.1101/gr.216902 PMC155283

