# Supplementary figures and images for: Expression of Human Endogenous Retrovirus-W Including Syncytin-1 in Cutaneous T-Cell Lymphoma
Source: PLoS One. 2013 Oct 1;8(10):e76281. doi: 10.1371/journal.pone.0076281 (PMC3788054; doi:10.1371/journal.pone.0076281)

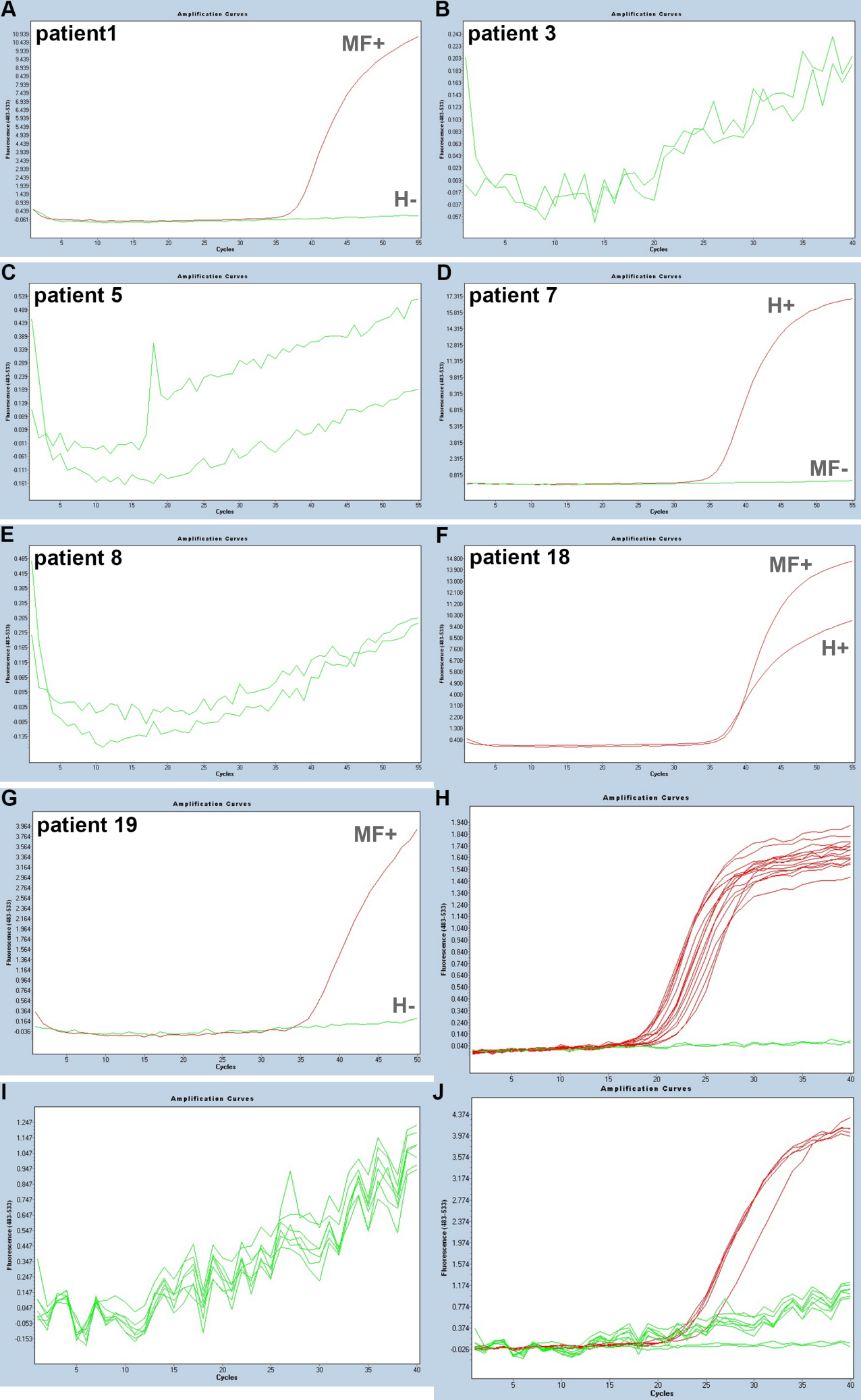

Supplement: Figure S1 — Amplification curves of studied MF and lichen ruber planus samples. A) ERVWE1 amplification in patient 1 (H-, MF+), B) no ERVWE1 amplification in patient 3, C) no ERVWE1 amplification in patient 5, D) ERVWE1 amplification in patient 7 (H+, MF-), E) no ERVWE1 amplification in patient 8, F) ERVWE1 amplification in patient 18 (H+, MF+), G) ERVWE1 amplification in patient 19 (H-, MF+), H) GAPDH amplification in all studied MF samples (red), also negative water controls are shown (green baseline), I) no ERVWE1 amplification in any of the Lichen ruber planus samples (n=5), and J) GAPDH (red) versus ERVWE1 (green) amplification of all studied Lichen ruber planus samples. Also negative water controls are presented (green baseline). H= clinically healthy, non-lesional, MF= MF lesion, + indicates amplification, - no amplification. (PDF) [file pone.0076281.s001.pdf]
