# Supplementary material for: Expression of Human Endogenous Retrovirus-W Including Syncytin-1 in Cutaneous T-Cell Lymphoma
Source: PLoS One. 2013 Oct 1;8(10):e76281. doi: 10.1371/journal.pone.0076281 (PMC3788054; doi:10.1371/journal.pone.0076281)
Supplement: Table S1 — Comparison of HERV core transcription pattern in human tissues. (PDF) [file pone.0076281.s002.pdf]

Table S1. Comparison of HERV core transcription pattern in human tissues.

HERV group	HERV subgroup	Skin	Urothelium [77]	Brain [60]	Mamma [63]	Kidney [39]
**HERV-FRD**	Seq46	-	-	-	+	-
**HERV-E**	HERV-E4-1	+	+	+	+	+
	Seq32	-	-	-	-	+
**HERV-F**	HERV-Fb	+	-	+	+	-
**HERV-Rb**	HERV-Rb	-	+	-	-	-
**HERV-W**	HERV-W	+	-	-	+	+
**ERV9**	Seq63	+	-	-	-	+
	ERV9	+	+	+	+	+
	Seq59	+	-	+	+	+
**HERV-K(HML-2)**	HERV-K10	-	-	-	+	+
	HERV-K (HML-2.HOM)	-	-	-	+	+
	HERV-K(HP1)	-	-	+	+	+
	HERV-K(D1.2)	-	-	+	+	+
**HERV-K(HML-3)**	Seq26	-	-	-	-	+
	HERV-K(HML-3)	-	-	-	+	+
**HERV-K(HML-4)**	HERV-K-T47D	+	+	+	+	-
	Seq10	-	-	+	+	-
**HERV-K(HML-6)**	HERV-K(HML-6)	-	-	+	+	+
	Seq38	-	-	+	+	-
	Seq56	-	-	-	+	-
**HERV-K(HML-8)**	NMWV3	-	+	-	-	+
**HERV-K(HML-9)**	NMWV9	-	-	+	-	-
**HERV-K(HML-10)**	HERV-KC4	-	+	+	-	-

“**+** “ indicates transcription and “–“ no transcription.
