# Supplementary material for: Expression of Human Endogenous Retrovirus-W Including Syncytin-1 in Cutaneous T-Cell Lymphoma
Source: PLoS One. 2013 Oct 1;8(10):e76281. doi: 10.1371/journal.pone.0076281 (PMC3788054; doi:10.1371/journal.pone.0076281)
Supplement: Table S2 — Summary of assignment of lesion and non-malignant skin tissue derived cDNA sequences to specific HERV-W loci. (PDF) [file pone.0076281.s003.pdf]

Table S2. Summary of assignment of lesion and non-malignant skin tissue derived cDNA sequences to specific HERV-W loci*.

				**total cDNAs**		**Patient 1**		**Patient 6**		**Patient 5**		**Patient 12**	
**Chr. band**	**HGNC symbol, aliases**	**tr. in PBMC**	**Location amplicon in hg18**	**Lesion**	**Non-malignant**	**Lesion**	**Non-malignant**	**Lesion**	**Non-malignant**	**Lesion**	**Non-malignant**	**Lesion**	**Non-malignant**
5q21.3	–		chr5:107939374-	0	9	0	9	0	0	0	0	0	0
			107939968	0.00	7.69	0.00	23.68	0.00	0.00	0.00	0.00	0.00	0.00
6q21	–	Y	chr6:106788519-	43	26	20	0	13	9	0	11	10	6
			106789158	37.07	22.22	64.52	0.00	37.14	32.14	0.00	37.93	45.45	27.27
7q21.2	ERVW-1 ERVWE1,	Y	chr7:91936835-	45	37	6	0	9	16	28	14	2	7
	Syncytin-1		91937430	38.79	31.62	19.35	0.00	25.71	57.14	100.00	48.28	9.09	31.82
11p14.3	–		chr11:22291012-	2	7	0	7	0	0	0	0	2	0
			22291605	1.72	5.98	0.00	18.42	0.00	0.00	0.00	0.00	9.09	0.00
12q13.13	–		chr12:49584194-	1	0	0	0	0	0	0	0	1	0
			49584790	0.86	0.00	0.00	0.00	0.00	0.00	0.00	0.00	4.55	0.00
15q21.3	ERVW-4, C187-23	Y	chr15:53385581-	6	7	0	0	6	2	0	4	0	1
			53386171	5.17	5.98	0.00	0.00	17.14	7.14	0.00	13.79	0.00	4.55
17q12	–	Y	chr17:32765922-	18	31	5	22	6	1	0	0	7	8
			32766547	15.52	26.50	16.13	57.89	17.14	3.57	0.00	0.00	31.82	36.36
Xq22.3	ERVW-2, ERVWE2	Y	chrX:106183224-	1	0	0	0	1	0	0	0	0	0
			106183878	0.86	0.00	0.00	0.00	2.86	0.00	0.00	0.00	0.00	0.00
				**116**	**117**	**31**	**38**	**35**	**28**	**28**	**29**	**22**	**22**

* Chr. band: chromosomal location of the transcribed HERV-W locus
HGNC symbol, aliases: Human Gene Nomenclature Committee approved HERV-W locus symbol if HERV locus has been identified as transcribed before and has been submitted as such to HGNC.
tr. in PBMC: "Y" indicates that that HERV-W locus has previously been identified as transcribed in PBMC [59].
Location amplicon in hg18": nucleotide position of RT-PCR amplicon on the respective human chromosome based on the hg18/March 2006 human reference sequence (NCBI Build 36.1).
total cDNAs: total number of cDNAs generated and assigned to the various HERV-W loci for "lesion tissue" and "non-malignant tissue". Numbers are also given in percentages based on the total number of analysed cDNAs (bottom of row) each.The following columns further detail results for the four different patients as described for "total cDNAs"
